# Novel use of a transapical endovascular suction device for mitral valve endocarditis in a high-risk surgical patient with successful cerebral protection

**DOI:** 10.1016/j.xjtc.2022.08.034

**Published:** 2022-10-13

**Authors:** Andrew P. Rabenstein, Adnan Khalif, David Lasorda, Rachel Hughes-Doichev, Michael Popeck, Mithun Chakravarthy, Walter McGregor

**Affiliations:** a Department of Thoracic and Cardiovascular Surgery, AHN Cardiovascular Institute at Allegheny General Hospital, Pittsburgh, Pa; b Department of Cardiology, AHN Cardiovascular Institute at Allegheny General Hospital, Pittsburgh, Pa

**Figure:**
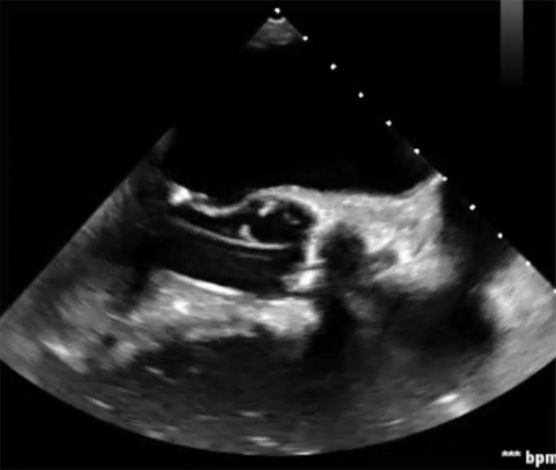
Echo-guided transapical suction aspiration of a mitral valve vegetation.

**Central Message**

We present a novel use of transapical suction aspiration with generous cerebral protection strategy for mitigation of embolic events in endocarditis.

## Case Report

### Patient Presentation

**Figure 1:**
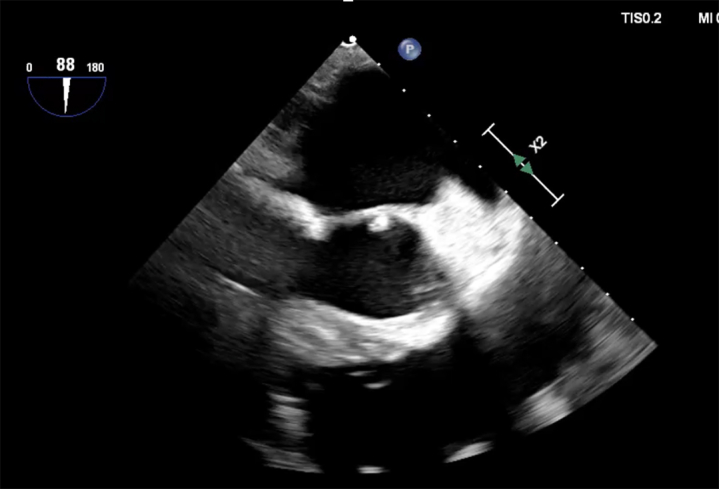
Echocardiographic demonstration of the mitral valve vegetation.

A 79-year-old man presented with a deep sternal wound infection 3 weeks after emergent repair of a ventricular perforation sustained during arrhythmia ablation. This was aggressively treated with sternal debridement and negative pressure wound therapy in preparation for plastic surgery reconstruction. Clinically, the patient progressed but began experiencing crescendo transient ischemic attacks. Echocardiography revealed a new mobile density at the base of the anterior mitral leaflet (Figure 1) as well as new small infarcts on imaging.

Traditional cardiac surgery was considered high risk because of: 1) severe left ventricular dysfunction, 2) moderate aortic and mitral valve disease (Videos 1 and 2), 3) a densely adherent and inflamed operating field, and 4) the need for a timely plastic surgical wound closure. We candidly offered standard of care mitral valve surgery,^1^ which the patient refused. We alternatively offered off-label AngioVac (Angiodynamics) use to remove the vegetation. The patient elected to proceed with off-label treatment.

### Procedure

This complex operation merged cardiac surgery, interventional cardiology, advanced echocardiography, and perfusion disciplines (The full procedure is available as Video 3). The left ventricular apex was exposed through a small left fifth interspace incision and secured with concentric pledgeted polypropylene purse-string sutures. Simultaneously, the right common femoral artery and vein along with the right radial artery were percutaneously accessed and the patient was heparinized to an activated clotting time of 250.

The Sentinel cerebral protection system (Boston Scientific)—a 2-stage embolic protection device—was inserted via the right radial artery and deployed into the innominate and left common carotid artery. An 8-French (F) occluding balloon was inserted via the right femoral artery and advanced to the ostium of the left subclavian artery to complete the cerebral protection strategy. This completed the positioning of our cerebral protection strategy (Video 4).

**Figure 2:**
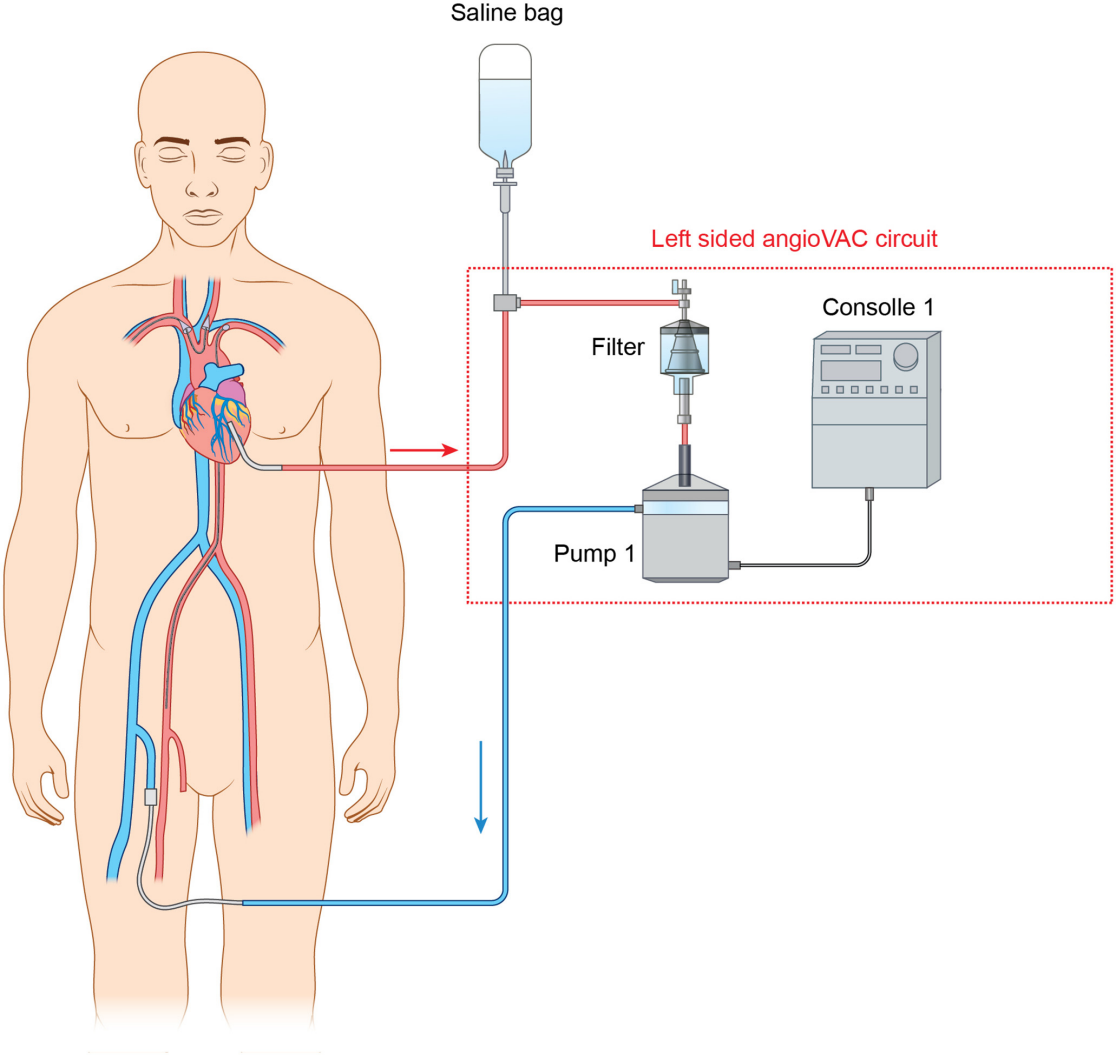
Diagram of the vascular access sites.

The left ventricular apex was accessed at a location where echocardiographic orthogonal planes predicted smooth passage of an Amplatz wire into the ascending aorta while avoiding the mobile vegetation and subvalvular mitral apparatus (Video 5). A 26-F sheath was placed through the ventricular apex and positioned at the vegetation. A 16-F venous return sheath was placed into the right femoral vein and the AngioVac circuit was completed (Figure 2) and deaired.

The AngioVac cannula was inserted through the transapical sheath and positioned close to the vegetation. The left subclavian balloon was inflated. The AngioVac was activated and navigated with echocardiography and fluoroscopy using multiple passes (Video 6) until adequate removal of the vegetation was confirmed (Video 7). Heparin was reversed and all sheaths and catheters were removed. The left ventricular apex purse strings were tied and the thoracotomy was closed in a standard fashion. The patient was transported to the intensive care unit. A postprocedural echocardiogram revealed 1-2+ mitral regurgitation (MR).

### Hospital Course

Postoperatively the patient did well and experienced no further neurologic sequelae. The patient's sternal wound was subsequently closed by Plastic Surgery and the patient was discharged to a rehabilitation facility with a 6-week course of cefazolin. He returned to home 1 month later.

The patient was serially followed by Internal Medicine and Cardiology. Progressive echocardiograms revealed increasing MR to 3-4+ MR. The patient refused additional interventions for his MR.

Approximately 4 months later the patient presented to the emergency room with symptoms of heart failure and expired in the emergency room shortly thereafter of unknown cause. His last echocardiogram was performed approximately 2 weeks before expiration and again revealed 3-4+ MR.

## Discussion

This case represents the second report^2^ of transapical suction aspiration of the mitral valve with the AngioVac device. The first case was performed by attaching the AngioVac circuit onto an extracorporeal membrane oxygenation circuit,^2^ whereas this case was without extracorporeal membrane oxygenation support.

This patient was clearly indicated for surgery via the 2016 consensus guidelines because of worsening endocarditis with embolization.^1^ However, the patient elected for only less invasive options than traditional cardiac surgery because of his high-risk profile. With our institution's experience with the AngioVac system as well as the aforementioned case report, we found it prudent to offer this treatment as an experimental option to the patient, with full disclosure of its novel and off-label use.

The key to performing this safely was the generous cerebral protection strategy. Although the AngioVac system uses negative pressure to minimize any embolization risk, by protecting the innominate artery and left common carotid artery with the Sentinel cerebral protection system and the left subclavian artery with balloon occlusion we were able to prevent any cerebral embolic phenomenon. The patient subsequently experienced no subsequent transient ischemic attacks. Examination of the Sentinel cerebral protection system revealed no appreciable solid material.

This lesion was accessible because of the ventricular-facing aspect. For atrial-facing vegetations or aortic-facing aortic valve vegetations this approach is less appealing. To date we have performed an off-label aortic valve aspiration for a mixed ventricular/aortic-facing vegetation with mixed results. This shows the importance of ascertaining which direction the vegetation is facing to appropriately frame an approach for a given vegetation.

Finally, it is noted that this technique is not the standard of care for normal endocarditis. Care should be taken to adhere to the consensus guidelines whenever possible. For patients for whom the operative risks are exceedingly high, AngioVac remains an off-label and yet to be fully explored option. This report shows the technical feasibility of mitral valve AngioVac via a transapical approach for embolic event risk reduction as part of a multidisciplinary heart team approach and patient/goal-directed care.

## Supplementary Data

Video 1 Echocardiogram showing mitral valve disease with mitral annular calcification. Video available at: https://www.jtcvs.org/article/S2666-2507(22)00530-2/fulltext.

Video 2 Echocardiogram showing mitral valve disease with mitral annular calcification. Video available at: https://www.jtcvs.org/article/S2666-2507(22)00530-2/fulltext.

Video 3 Complete case video. Video available at: https://www.jtcvs.org/article/S2666-2507(22)00530-2/fulltext.

Video 4 Final positioning of the cerebral protection strategy. Video available at: https://www.jtcvs.org/article/S2666-2507(22)00530-2/fulltext.

Video 5 Loading the transapical AngioVac (Angiodynamics) system. Video available at: https://www.jtcvs.org/article/S2666-2507(22)00530-2/fulltext.

Video 6 Active suction aspiration of the vegetation. Video available at: https://www.jtcvs.org/article/S2666-2507(22)00530-2/fulltext.

Video 7 Completion echocardiogram. Video available at: https://www.jtcvs.org/article/S2666-2507(22)00530-2/fulltext.
