# Supplementary figures and images for: Novel use of a transapical endovascular suction device for mitral valve endocarditis in a high-risk surgical patient with successful cerebral protection
Source: JTCVS Tech. 2022 Oct 13;16:60–3. doi: 10.1016/j.xjtc.2022.08.034 (PMC9737037; doi:10.1016/j.xjtc.2022.08.034)

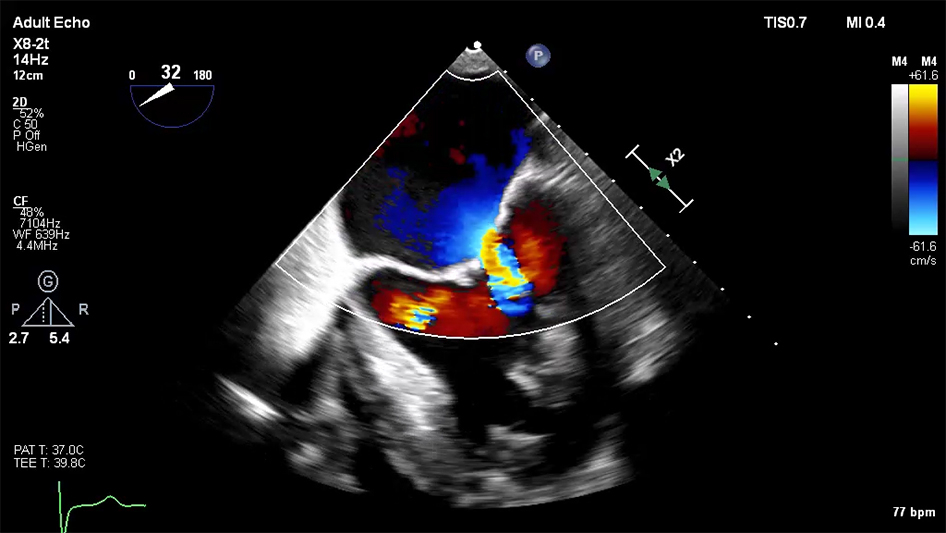

Supplement: Video 1 — Echocardiogram showing mitral valve disease with mitral annular calcification. Video available at: https://www.jtcvs.org/article/S2666-2507(22)00530-2/fulltext. [file fx2.jpg]

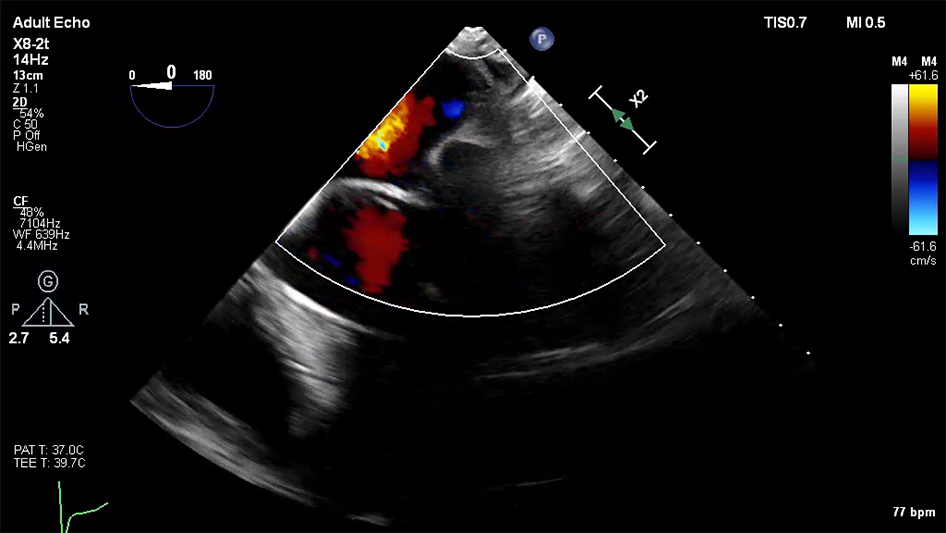

Supplement: Video 2 — Echocardiogram showing mitral valve disease with mitral annular calcification. Video available at: https://www.jtcvs.org/article/S2666-2507(22)00530-2/fulltext. [file fx3.jpg]

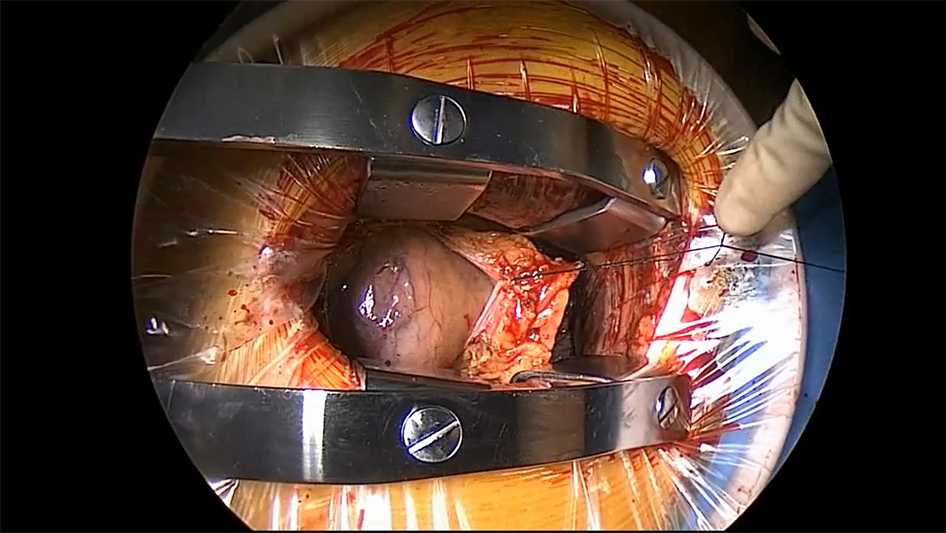

Supplement: Video 3 — Complete case video. Video available at: https://www.jtcvs.org/article/S2666-2507(22)00530-2/fulltext. [file fx4.jpg]

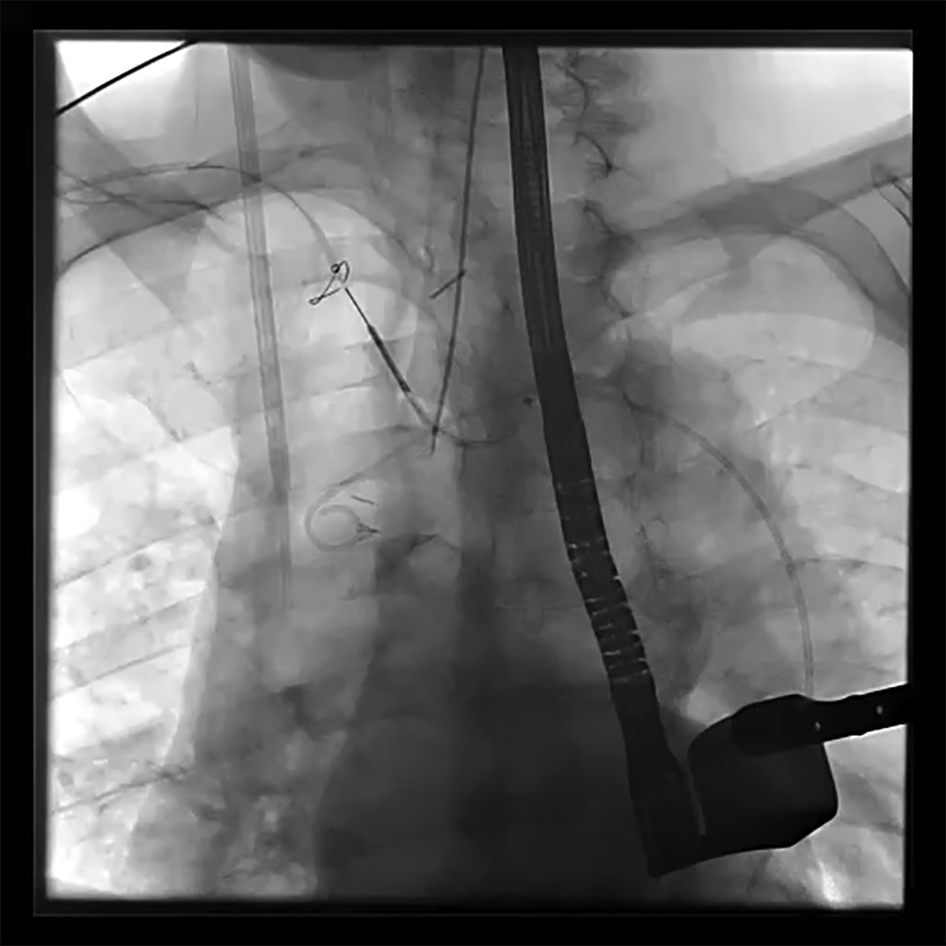

Supplement: Video 4 — Final positioning of the cerebral protection strategy. Video available at: https://www.jtcvs.org/article/S2666-2507(22)00530-2/fulltext. [file fx5.jpg]

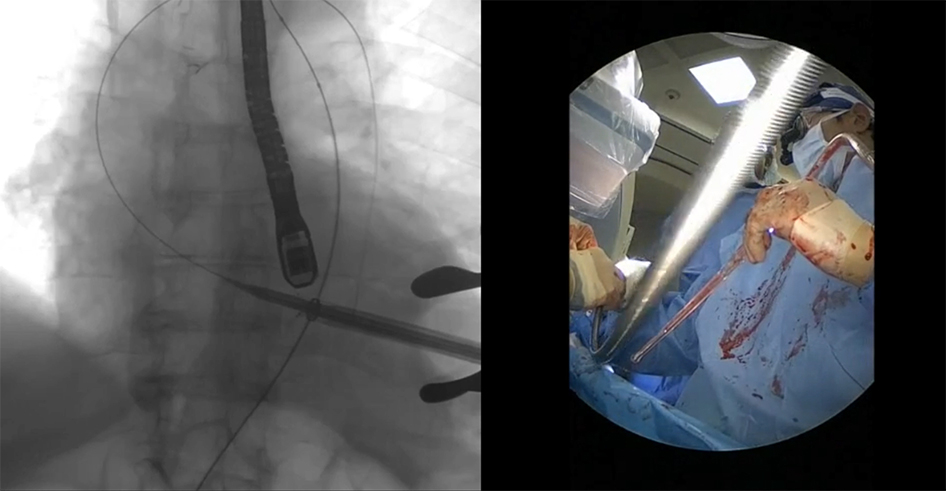

Supplement: Video 5 — Loading the transapical AngioVac (Angiodynamics) system. Video available at: https://www.jtcvs.org/article/S2666-2507(22)00530-2/fulltext. [file fx6.jpg]

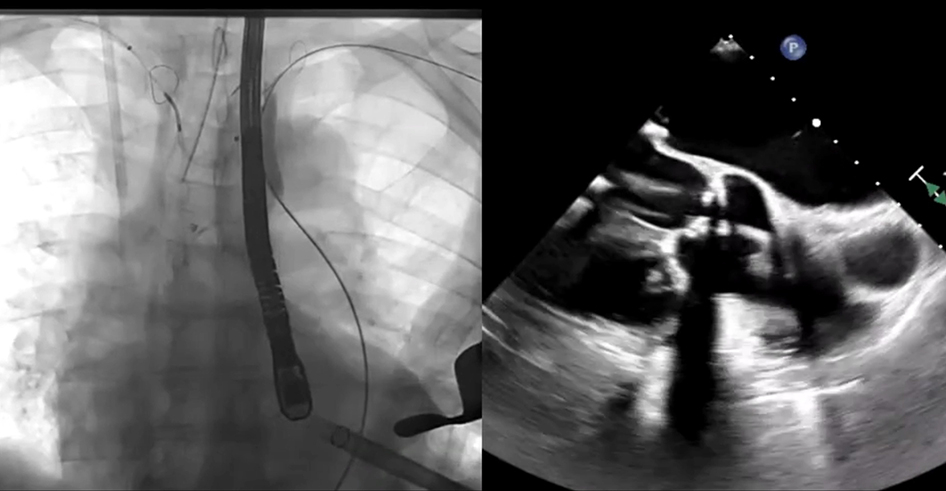

Supplement: Video 6 — Active suction aspiration of the vegetation. Video available at: https://www.jtcvs.org/article/S2666-2507(22)00530-2/fulltext. [file fx7.jpg]

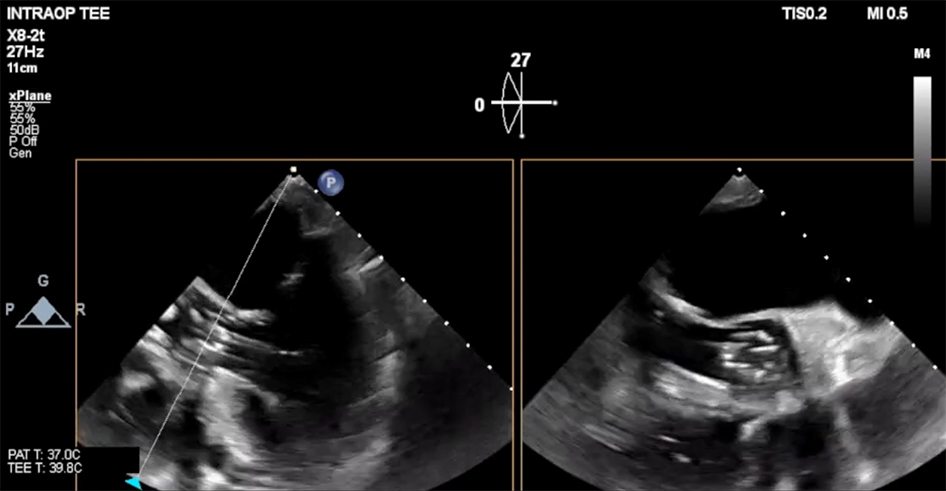

Supplement: Video 7 — Completion echocardiogram. Video available at: https://www.jtcvs.org/article/S2666-2507(22)00530-2/fulltext. [file fx8.jpg]
